# Imaging functional neuroplasticity in human white matter tracts

**DOI:** 10.1007/s00429-021-02407-4

**Published:** 2021-11-23

**Authors:** Tory O. Frizzell, Elisha Phull, Mishaa Khan, Xiaowei Song, Lukas A. Grajauskas, Jodie Gawryluk, Ryan C. N. D’Arcy

**Affiliations:** 1 BrainNET, Health and Technology District, Surrey, BC Canada; 2 Faculty of Applied Sciences and Science, Simon Fraser University, Vancouver, BC Canada; 3 Health Sciences and Innovation, Surrey Memorial Hospital, Surrey, BC Canada; 4 Cumming School of Medicine, University of Calgary, Calgary, AB Canada; 5 Division of Medical Sciences, Department of Psychology, University of Victoria, Victoria, BC Canada; 6 DM Centre for Brain Health (Radiology), University of British Columbia, Vancouver, BC Canada

**Keywords:** Diffusion tensor imaging, Functional magnetic resonance imaging, Low-frequency oscillations, Functional correlation tensors, White matter activation

## Abstract

Magnetic resonance imaging (MRI) studies are sensitive to biological mechanisms of neuroplasticity in white matter (WM). In particular, diffusion tensor imaging (DTI) has been used to investigate structural changes. Historically, functional MRI (fMRI) neuroplasticity studies have been restricted to gray matter, as fMRI studies have only recently expanded to WM. The current study evaluated WM neuroplasticity pre–post motor training in healthy adults, focusing on motor learning in the non-dominant hand. Neuroplasticity changes were evaluated in two established WM regions-of-interest: the internal capsule and the corpus callosum. Behavioral improvements following training were greater for the non-dominant hand, which corresponded with MRI-based neuroplasticity changes in the internal capsule for DTI fractional anisotropy, fMRI hemodynamic response functions, and low-frequency oscillations (LFOs). In the corpus callosum, MRI-based neuroplasticity changes were detected in LFOs, DTI, and functional correlation tensors (FCT). Taken together, the LFO results converged as significant amplitude reductions, implicating a common underlying mechanism of optimized transmission through altered myelination. The structural and functional neuroplasticity findings open new avenues for direct WM investigations into mapping connectomes and advancing MRI clinical applications.

**Supplementary Information:**

The online version contains supplementary material available at 10.1007/s00429-021-02407-4.

## Introduction

The human brain is a dynamic and highly integrated system with a dense array of connections, which in essence, allows for performance of tasks and behavioral change. White matter (WM), which is composed of axons and associated glial cells (e.g., oligodendrocytes that produce myelin), connects different brain regions and makes up approximately 50% of brain tissue (Sampaio-Baptista and Johansen-Berg 2017).

Importantly, WM changes are experience-dependent and have been well established as a critical contributing aspect of neuroplasticity in the adult brain (Sampaio-Baptista and Johansen-Berg 2017; Fields 2011). Specifically, activity-dependent regulation of myelin through oligodendrocytes and oligodendrocyte-precursor cells appear to play an important role in both structural and functional neuroplasticity (Sampaio-Baptista and Johansen-Berg 2017; Foster et al. 2019). During learning, for instance, experience-dependent WM neuroplasticity alters structural axon properties, such as myelin, axon diameter, and internode length, leading to functional and physiological changes such as conduction speed (Fields 2015; Sampaio-Baptista and Johansen-Berg 2017). Notably, much of the in vivo evidence of WM neuroplasticity has come from magnetic resonance imaging (MRI) studies. The first MRI investigation of experience-dependent changes in WM was published by Scholz et al. (2009), who demonstrated diffusion tensor imaging (DTI) measured increases in fractional anisotropy (FA) following 6 weeks of training on a visuomotor task (juggling) (Scholz et al. 2009). In the decade since this original study, the majority of WM neuroplasticity studies have focused on changes in microstructure using gold standard DTI metrics (Reid et al. 2017; Sampaio-Baptista and Johansen-Berg 2017; Deng et al. 2018; Hamaide et al. 2016). Although DTI can provide valuable information about neuroplasticity changes in WM microstructure, it does not measure functional neuroplasticity.

Functional MRI (fMRI) studies have used blood-oxygen level-dependent (BOLD) contrast to track gray matter (GM) neuroplasticity changes in healthy individuals during learning (Sale et al. 2017; Chang 2014; Keller and Just 2016), in task-related recovery of function (Tombari et al. 2004; DʼArcy et al. 2016), and in resting state network changes [e.g., (Lewis et al. 2009)]. For instance, Sale et al. (2017) showed that motor training of participants’ non-dominant (left) hands led to detectable behavioral performance improvements along with corresponding GM neuroplasticity changes, as measured by fMRI and cortical thickness (Sale et al. 2017). In a follow-up study, Reid et al. (2017) conducted fMRI-guided diffusion MRI and showed significant FA increases within the right corticospinal tract along with associated WM tracts (Reid et al. 2017). The authors further evaluated WM neuroplasticity using transcranial magnetic stimulation (TMS) and demonstrated increased motor-evoked potentials to infer functional gains along the corticospinal tract. Taken together, Reid et al. (2017) postulated that the multimodal imaging results supported an increase in myelination as a result of the motor training task.

To date, these landmark studies have demonstrated WM changes in microstructure as a consequence of motor task training and evidence of motor dependent functional neuroplasticity in GM. However, direct fMRI activation changes related to WM neuroplasticity have been a critical missing gap.

Foundational work on WM fMRI has demonstrated the utility of the technique to measure activation and functional connectivity within WM tracts (Gawryluk et al. 2014). WM activation in fMRI enabled a perspective shift in functional mapping of distributed neural networks (Grajauskas et al. 2019). Specifically, rather than WM connectivity inferences indirectly relying on changes in GM activation within connectomes, the reverse became possible: localized changes in WM activation can be directly examined, and then linked to GM activation. Consequently, these localized WM changes can be experimentally manipulated through functional neuroplasticity. In particular, WM BOLD responses have been robustly captured in the corpus callosum. The anterior body of the corpus callosum has been repeatedly linked with activation as a result of motor and visuomotor tasks (Weber et al. 2005; Tettamanti et al. 2002; Fabri et al. 2011), while other regions of the corpus callosum have exhibited activity in response to various visual, tactile, and interhemispheric transfer tasks (Fabri 2014; Fabri et al. 2011; Mazerolle et al. 2008, 2010; Steele et al. 2013; Mishra et al. 2020; D’Arcy et al. 2006). There are also increasing reports of WM activation in the internal capsule (Frizzell et al. 2020; Gawryluk et al. 2011a, b; Mosier et al. 1999; Maldjian et al. 1999). These studies have confirmed involvement of the corticospinal tract directly with fMRI activation detected across a range of different motor tasks. Interestingly, Vien et al. (2016) also found that there was a positive correlation between FA in the internal capsule as well as the corpus callosum with performance during a motor learning training session.

Frizzell et al. (2020) recently used BOLD fMRI to examine WM neuroplasticity within a longitudinal motor learning task similar to the prior non-dominant hand training experiments (Reid et al. 2017; Sale et al. 2017). In the study, participants underwent 2 weeks of motor training using both their non-dominant and dominant hands, with MRI acquisition at the start, middle, and end points. As with the prior work, significant behavioral performance improvements were detected for the non-dominant (left) hand between baseline and endpoint evaluations. Corresponding to the behavioral results, BOLD fMRI detected significant changes in WM hemodynamic response within the right internal capsule (i.e., left hand). Initial examination of the BOLD hemodynamic response function (HRF) within the internal capsule revealed a significant reduction in HRF signal variability (Frizzell et al. 2020). This finding provided potential support for the concept that neuroplasticity in WM reflects changes in transmission efficiency (i.e., an efficiency hypothesis). With respect to underlying mechanisms related to increased and/or altered myelination in WM neuroplasticity, the reduced HRF variability implicated increased functionally correlated activation within the internal capsule.

Consequently, this work led to two critical aims that remain to be addressed:

*Aim 1*: Improved WM fMRI sensitivity: convergent fMRI evidence of functional neuroplasticity:

First, emerging MRI methods can improve WM fMRI sensitivity to functional neuroplasticity (Aim 1). In particular, newer methods, including the measurement of low-frequency oscillations (LFOs) or functional correlation tensors (FCTs) may provide unique information about WM functional changes.

LFOs represent intrinsic neural oscillations typically less than 0.1 Hz as detected from BOLD fMRI time series data (Biswal et al. 1995). LFOs are believed to be responsible for recruiting and synchronizing brain networks and neural and cognitive processes (Buzsáki and Draguhn 2004). They are task-modulated and have been studied extensively in GM for the past 25 years (Niu et al. 2014; Biswal et al. 1995; Zhan et al. 2016; Fryer et al. 2016; Zuo et al. 2010; Xu et al. 2006), but have only recently been observed in specific WM structures (Ji et al. 2017; Wu et al. 2016). For instance, Jiang et al. (2021) found that schizophrenic patients had higher amplitude of low-frequency fluctuation (ALFF; an index of LFO amplitude) in WM structures (e.g., corona radiata, internal capsule, and corpus callosum) compared to healthy controls, which was indicative of network disruption, alluding to the role of LFO amplitude in efficiency of networks and connectivity.

While relatively new, FCTs use a mathematical approach similar to DTI to analyze local correlation in BOLD signals. With increasing evidence of WM hemodynamic response variability (Li et al. 2019, 2021; Wang et al. 2020a, b), FCT provides an alternative measure sensitive to potentially undetectable WM activation using conventional fMRI analyses. Specifically, FCT does not rely on hemodynamic response functions or thresholding that may dampen the comparatively weaker signals of WM BOLD and can be decomposed into metrics commonly used in DTI analyses such as FA (Ding et al. 2013; Zhang et al. 2017). The convergent relationship in WM fMRI between FCTs and LFOs has been previously examined in cross section during non-human primate research (Wu et al. 2016)^.^ Consequently, an important next step involves human brain mapping of WM functional neuroplasticity using both LFO and FCT approaches.

*Aim 2*: functional MRI changes can be linked back to DTI structural changes:

Second, it is imperative that the measured functional changes in WM be linked back to the underlying structural changes, using gold standard DTI methods (Aim 2). To date, DTI changes resulting from motor training have provided initial evidence of a structure–function link (e.g., Reid et al. 2017; Sale et al. 2017). Recent comparisons between DTI and FCT tensors have further enabled examination of the relationship between structural and functional changes, with demonstration that FCT findings closely follow DTI tractography (Zhang et al. 2017; Ding et al. 2016). Likewise, LFO amplitudes have been positively linked with FA (Ji et al. 2017). However, a gap in the literature that must still be addressed is the relationship between functional neuroplasticity measured by WM fMRI, and microstructural plasticity in white matter, measured by DTI.

Based on the accumulating MRI imaging evidence to date, the objective of the current study was to specifically evaluate WM fMRI neuroplasticity during motor learning in two key regions-of-interest (ROIs): (1) the internal capsule; and (2) the corpus callosum. These ROIs represent established active regions within the motor network of interest, providing a foundational starting point given that they are known to be detectable and involved in motor learning. Within these target ROIs, WM neuroplasticity changes were evaluated across MRI measures: DTI-FA, BOLD-fMRI, LFOs, and FCTs. Non-dominant greater than dominant hand motor performance changes were evaluated across all MRI measures. The hypothesis predicted convergent WM neuroplasticity effects due to common sensitivities to underlying myelination changes. With converging MRI evidence, the findings would lend strong support to the emerging theory of transmission efficacy optimization as an underlying mechanism in WM neuroplasticity. Directly measurable by MRI, metrics for increased transmission efficiency have the potential to significantly impact characterization of connectomes and clinical neuroplasticity in the human brain.

## Methods

### Participants

Thirty-six (36) scans were acquired in a longitudinal study of 12 healthy participants (seven females) over three time points (baseline, midpoint, and endpoint). All participants (age: 25.8 ± 3.7 years), were right-hand-dominant with normal or corrected to normal vision, and no history of neurological illness. The study was approved by the Research ethics boards of Simon Fraser University, the Fraser Health Authority, and the University of British Columbia. Written informed consent was provided by all participants prior to data collection.

### Experimental design

A 2-week motor-training task was completed by all participants, which was developed based on prior experiments (Reid et al. 2017; Sale et al. 2017). While a detailed account of current experimental design is provided by Frizzell et al. (2020), a brief summary description follows. Participants trained on a visual–motor task that required accurate and time-dependent tracing through a complex path displayed on a screen. Each participant was scanned at baseline, after 1 week of motor control training, and at endpoint after completing 2 weeks of motor control training. During the MRI sessions, participants completed a fine motor task during two separate fMRI scans—once with their non-dominant hand and once with their dominant hand. The order of the fMRI tasks was randomized during each session. Each functional scan was 6-min long with seven task active blocks of 24 s interleaved with jittered rest blocks averaging 24 s. The rest blocks showed a blank screen with a fixation cross, while the task blocks displayed a unique, randomized trial. During the task blocks, participants used an MRI compatible mouse to guide their cursor through the displayed trail with the appropriate hand. All participants did identical motor control training using both their non-dominant (left) and dominant (right) hands to examine training effect differences between the left and the right hands (i.e., left > right). Participants performed a training task daily at home as well as during the 3 MRI time points. Similar to the MRI scan sessions, each training session, the participants completed a training task once with their non-dominant hand and once with their dominant hand (random order). Motor control performance improvements were evaluated based on a speed-accuracy metric to confirm left > right-hand learning effects.

### MRI acquisition

All MRI data were acquired with a 3-Tesla Philips INGENIA CX MRI scanner with a 32-channel dStream head coil. BOLD-fMRI data were collected using single-shot gradient echo type echo planar imaging. The acquisition parameters were as follows: TR = 2000 ms, TE = 30 ms, and flip angle = 90°. During the same session, DTI data were acquired using a single-shot EPI sequence with 32 diffusion directions and b-value of 800. To provide co-registration of functional images, 3D high-resolution T1-weighted images were also acquired. Scan acquisition parameters were as follows: TR = 8.2 ms, TE = 3.7 ms, and flip angle = 8°.

### Aim 1: Improved WM fMRI sensitivity: convergent fMRI evidence of functional neuroplasticity

#### BOLD pre-processing

All fMRI data were pre-processed using FSL v.6.0.0 BET and FEAT functions following standard procedures (Smith 2002; Woolrich et al. 2001). Brain extraction was done using the BET function and motion correction was completed using the MCFLIRT function. The fMRI data were also slice time corrected and temporally high-pass-filtered with a cut-off of 100 s (0.01 Hz, LFO sub-bands above high-pass cut-off, see below). No spatial smoothing was used to avoid introducing false spatial–temporal correlation. Functional MRI images were registered to the high-resolution T1WI using FSL’s FLIRT function and then warped to the MNI152 template using the FNIRT function.

#### ROI and HRF extractions

The internal capsule and the body of the corpus callosum were selected as ROIs based on prior literature (Fabri 2014; Fabri et al. 2011; Steele et al. 2013; Mishra et al. 2020; Mazerolle et al. 2008; D’Arcy et al. 2006; Gawryluk et al. 2011a, b; Mosier et al. 1999; Maldjian et al. 1999; Mazerolle et al. 2010). ROIs were extracted using the FSL brain atlas for the FCT, DTI, and WM HRF analyses. The LFO analysis subsequently focused on the WM HRF and FCT activity within each ROI. White matter-specific HRFs followed prior methods (Frizzell et al. 2020; Courtemanche et al. 2018), incorporating a delayed-onset slope and a reduced initial overshoot to approximate task-based WM HRFs. FSL toolkit “FMRIB’s Linear Optimal Basis Sets” (FLOBS) was used to generate an optimal basis set of three bases: an HRF curve, a latency derivative, and a dispersion derivative. Individual and group level analyses were computed using FSL’s FEAT function (*z* > 2.5, *p* < 0.05, two-tailed, FWE). Group level analyses revealed a significant decrease in the dispersion derivative within the internal capsule from baseline to endpoint for the non-dominant hand task condition. The internal capsule ROI mask was computed using this significant cluster. The corpus callosum ROI mask was similarly computed using the FCT analyses described in Sect. 2.7 below. Right-hand control comparisons used homologous ROIs mirrored to the contralateral hemisphere.

#### LFO analysis

For all of the ROIs, average BOLD voxel intensity was extracted as time series data from the pre-processed fMRI scans for both the non-dominant and dominant hand task data.

The time series data were demeaned. Using MATLAB’s Fast Fourier Transform (FFT) function, data were converted to frequency domain.

Following Jiang et al. (2019), two frequency bands for LFO analysis were selected. An additional band below the TR = 2000 ms Nyquist frequency was analyzed to capture any other low-frequency oscillation results. The following bands were examined: Band A (0.01–0.08 Hz); Band B (0.08–0.15 Hz); and Band C (0.15–0.22 Hz), whole band (0.01–0.22 Hz). For each frequency band, the average amplitude was computed for each participant at baseline and endpoint.

The effect of training between baseline and endpoint on frequency band amplitude was investigated using a heteroskedastic linear mixed-effects model in R Studio. Participant was set as the random-effect; whereas, ROI and frequency bands as fixed-effects. The model was adjusted for correlation of repeated observations.

Bivariate Pearson correlations were computed between each pair of LFOs within and between each WM ROI using MATLAB’s corr function in a pairwise manner with a two-tailed hypothesis test of no correlation against the alternative hypothesis of a non-zero correlation. The correlations at baseline, and endpoint were determined separately. The R function *corrplot* was used to visualize the significant correlations (*p* < 0.05, two-tailed).

#### FCT analysis

The FCTs were computed using the pre-processed fMRI data without spatial smoothing and individual tissue probability maps computed from participant high-resolution T1WI images for both GM and WM. Tissue probability maps were computed using FSLs FAST segmentation tool. A MATLAB script, adapted from Zhou et al. (2018) existing code for patch-based FCT (https://github.com/zyjshmily/ts-PFCTs), was used to compute the spatio-temporal tensors, and the resultant FCT FA maps for each fMRI scan. FCT FA maps were computed for each participant for both the non-dominant and dominant hand task data at baseline as well as endpoint. FSL’s tract-based spatial statistics (tbss) function was used to correct and register the resulting FCT FA maps to the MNI152 standard space. Group level statistics were set up using FSL’s GLM function for pairwise comparisons between baseline and endpoint. Significance maps were computed using FSL’s randomize function using threshold free cluster enhancement (*p* < 0.05, two-tailed, FWE).

### Aim 2: functional MRI changes can be linked back to DTI structural changes

#### DTI analysis

DTI data were analyzed using FSL v6.0.0 Diffusion Toolkit following standard procedures (Smith et al. 2004). The data were motion and eddy current corrected (Andersson and Sotiropoulos 2015). Diffusion tensors and dependent metrics including FA were calculated using FSL’s DTIFIT function. FSL’s tbss were used to correct and register FA maps to a standard space. All FA maps were projected onto a WM skeleton using a standard threshold of 0.2 to ensure exclusion of GM and CSF voxels.

The same FSL atlas ROIs were used as in the FCT analysis; the body of the corpus callosum and internal capsule. Significant group level effects between the endpoint and baseline were experimentally tested using homologous ROIs in the other hemisphere. Group level statistics were set up using FSL’s Glm function pairwise between endpoint and baseline. Significance maps were computed using FSL’s randomize function using threshold free cluster enhancement (*p* < 0.05, two-tailed, FWE).

#### Structure–function correlation analysis

To maximize comparability across modalities; FCTs were registered using FSL DTI specific analyses pipelines. Correlation analysis was computed using the participants DTI FA and FCT FA voxel intensities, masked to the aforementioned FSL atlas corpus callosum body ROI. Pearson's correlation coefficients were computed between the DTI structural metrics and the functional FCT metrics using MATLAB’s correlation coefficient function. RStudio’s *cocor* function was used for comparing correlation differences between baseline and endpoint for dependent groups, with non-overlapping measures, two-tailed, and alpha level of 0.05.

## Results

**Fig. 1:**
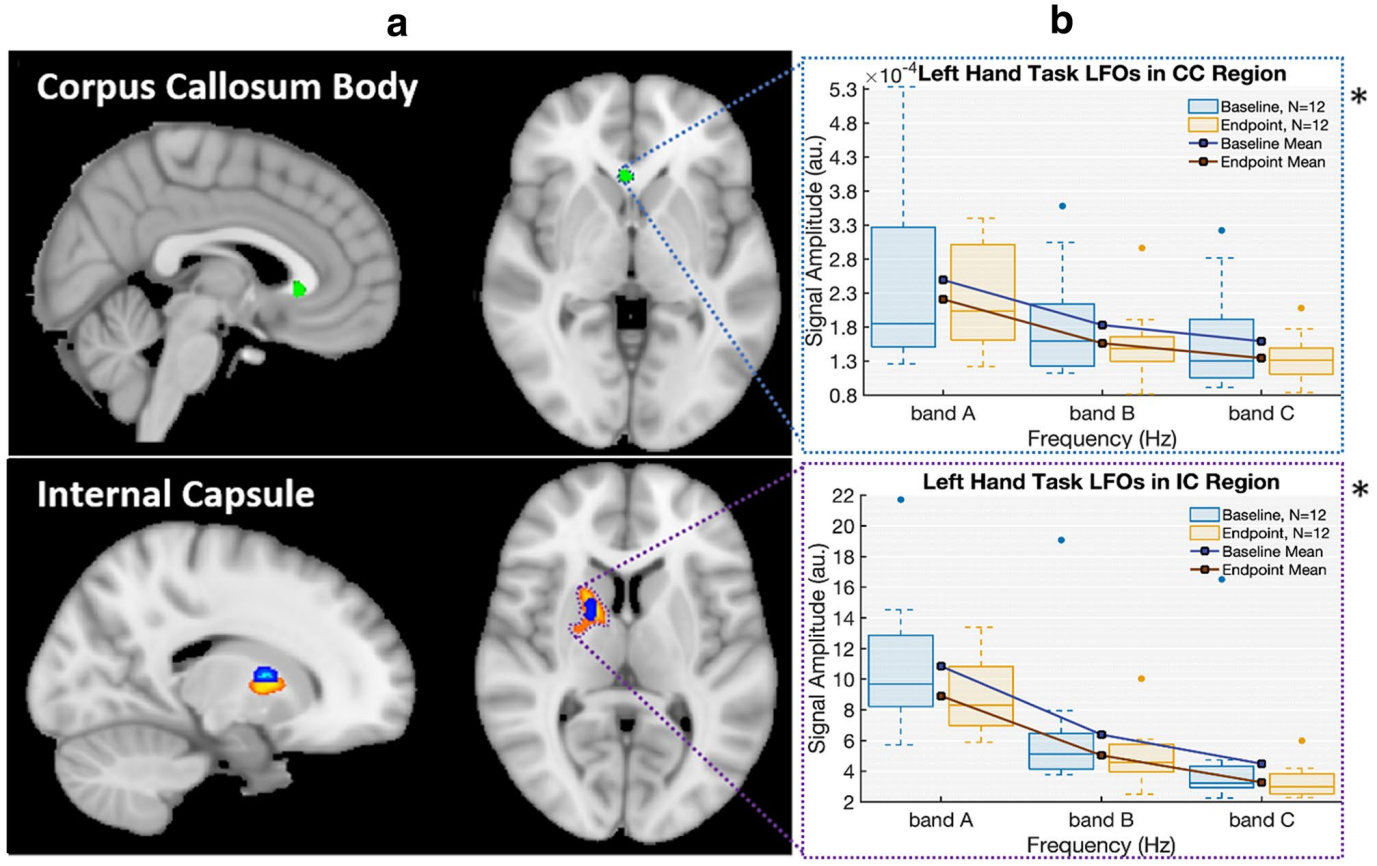
Evidence of motor learning driven neuroplasticity in white matter regions-of-interest. Panel A: summary of ROI results across: (1) DTI—blue; significant increase in DTI FA (baseline < endpoint; *p* < 0.05, FWE); (2) WM-HRF—orange; significant decrease in HRF dispersion derivative (baseline > endpoint; *p* < 0.05, FWE); and (3) FCT—green; significant increase in FCT FA (baseline < endpoint; *p* < 0.05, FWE). Panel B: convergence of LFO results, with graphs showing common significant amplitude decreases across frequency bands (baseline > endpoint; *p* < 0.05) with interquartile ranges

Examination of the WM ROIs for the non-dominant (left) hand showed significant changes in BOLD fMRI and DTI in the corresponding right internal capsule along with significant FCT changes in the anterior body of the corpus callosum (Fig. 1, panel A). FCT results demonstrated increased sensitivity beyond the internal capsule to initially identify functional neuroplasticity effects in the corpus callosum. Importantly, LFOs showed a common result across both ROIs. Specifically, there was a significant LFO amplitude reduction that was common in both the corpus callosum and internal capsule and across multiple frequency bands (Fig. 1, panel B). Consistent with prior results, lower-frequency bands (i.e., band A) had the largest amplitudes (Jiang et al. 2019; Biswal et al. 1995).

**Fig. 2:**
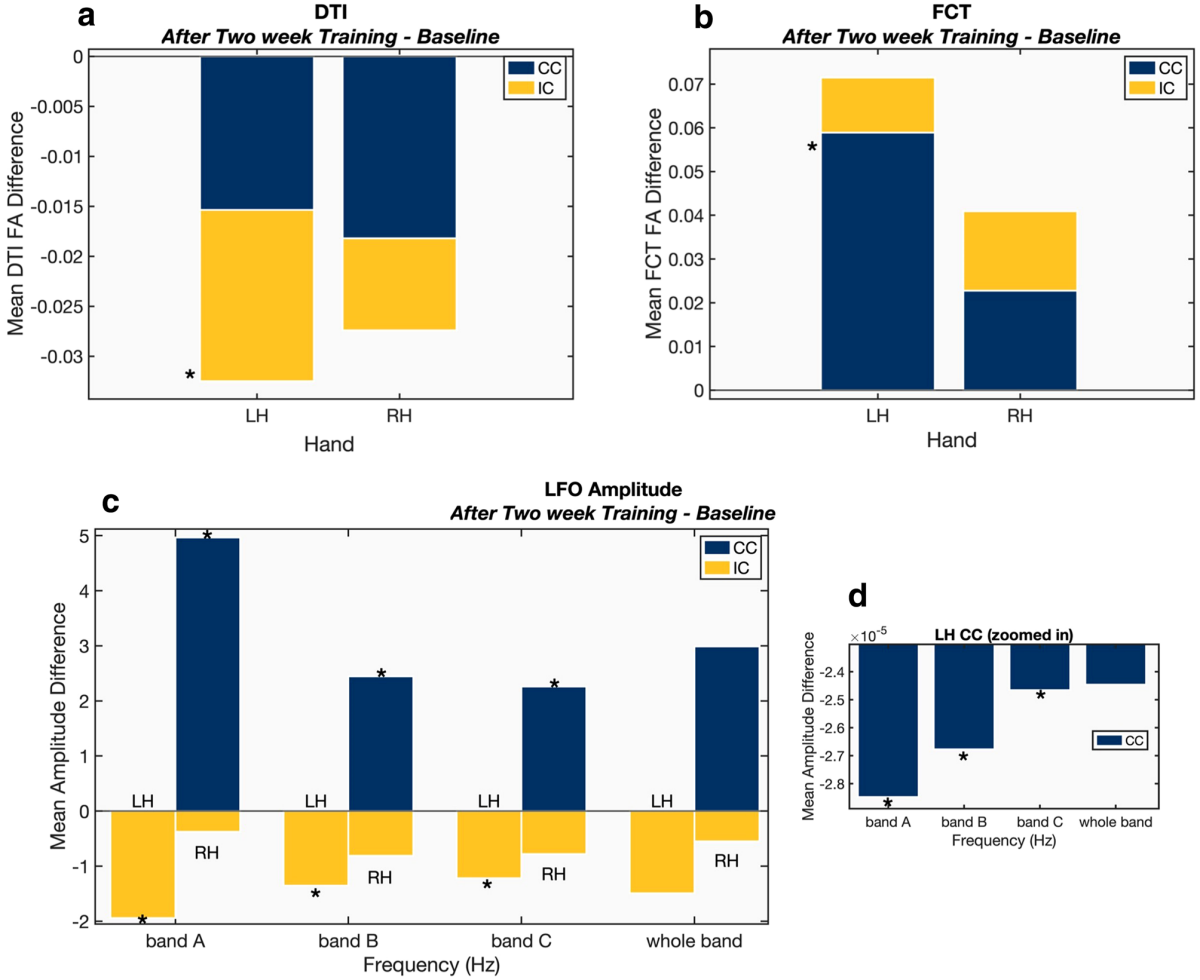
Endpoint—baseline neuroplasticity effects within CC and IC ROIs. Panel A—DTI FA, Panel B—FCT FA, Panel C & D—LFOs. *Significant group level differences between baseline and endpoint. DTI & FCT (*p* < 0.05, FWE) and LFO (p < 0.05). Whole bands (bands A through C, inclusive) were not included in the statistical analysis. [*LH* left hand task (right hemisphere ROIs), *RH* right-hand task (left hemisphere ROIs), *CC* corpus callosum ROIs, *IC* internal capsule ROIs]

**Fig. 3:**
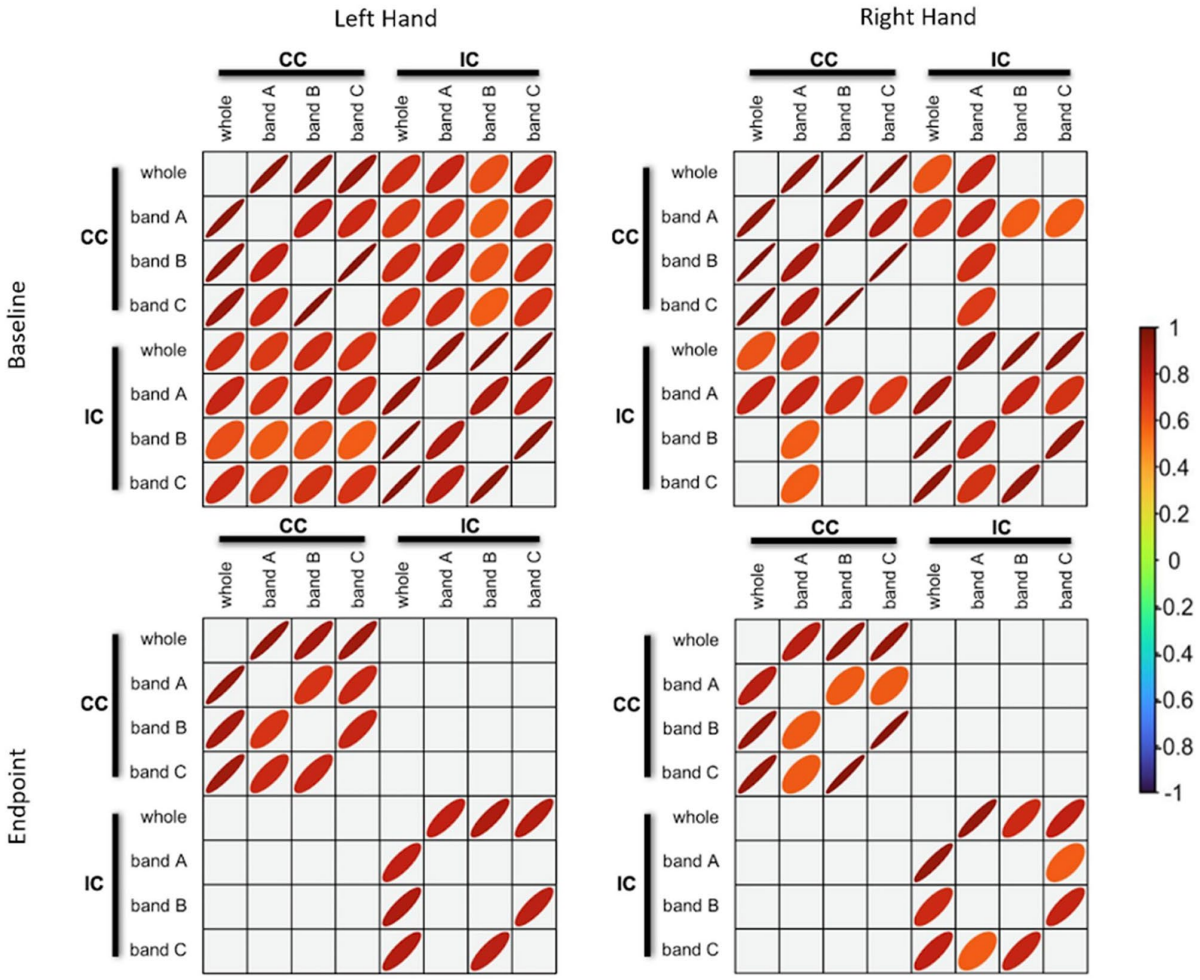
LFO Correlational analysis**.** Significant Bivariate pairwise Pearson correlations for each WM ROI LFO at baseline and after 2-week training (*p* < 0.05). The thickness of the ellipsoid indicates magnitude of the relationship, such that a thin ellipsoid represents a stronger correlation value (closer to ± 1); whereas a rounder ellipsoid represents weaker correlation. The color of the ellipsoid indicates the sign (positive, negative, no correlation) of the relationship between two variables. A positive relationship is red, negative is blue, and no correlation is green. Whole band: 0.01–0.22 Hz; band A: 0.01–0.08 Hz; band B: 0.08–0.15 Hz; band C: 0.15–0.22 Hz. (*CC* corpus callosum ROI, *IC* internal capsule ROI)

For experimental comparison, results for the non-dominant (left) hand were compared to those from the dominant (right) hand. Similar to behavioral motor performance results, the left greater than right asymmetry was observed across both ROIs, with one notable exception (below). As expected, significant left > right hand (LH > RH) differences were detected for DTI in the internal capsule (Fig. 2, panel A). Significant LH > RH differences were also present for FCT in the corpus callosum (Fig. 2, panel B). Similarly, examination of the LFO findings showed significant LH > RH differences in the internal capsule (Fig. 2, panel C). However, the reverse pattern of RH > LH differences was observed for the corpus callosum (Fig. 3).

Given the common LFO amplitude results across the ROIs, correlational analyses were conducted to evaluate the relationship between ROIs, baseline and endpoint, and response hand (Fig. 3). Examination of correlation matrices revealed an overall reduction in cross-correlations from baseline to endpoint, with a LH > RH difference. At endpoint, only intra-correlations were detected for the internal capsule and corpus callosum ROIs.

**Fig. 4:**
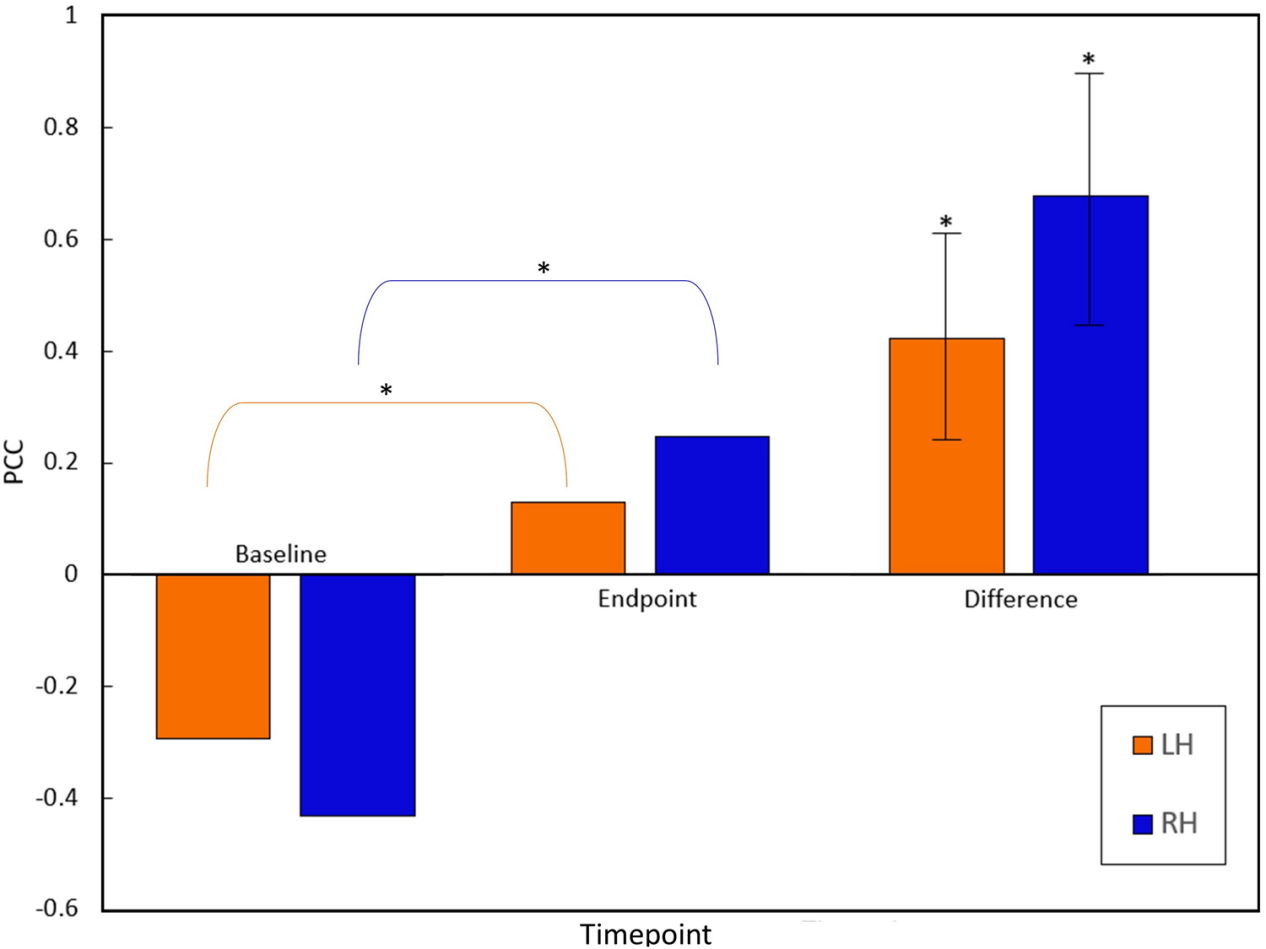
DTI FA-FCT FA correlational analysis. Significant DTI-FCT increases in Pearson correlations for the corpus callosum between baseline and endpoint. Results are shown for the left and right hands, significant differences between time points (*p* < 0.05) with standard error

To further investigate the structure–function relationship in the corpus callosum, correlational analyses were also conducted to explore the relationship between DTI and FCT results. The group mean DTI FA and FCT FA were computed for all participants to verify overall correspondence in WM tissue. Within the corpus callosum ROI, there were significant increases in the correlations between DTI and FCT between baseline and endpoints for both the left and right hands (Fig. 4). Pearson’s correlation coefficient increased significantly from baseline to endpoint for both the left- and right-hand tasks (*r* = 0.6 and 0.4 respectively, *p* < 0.05).

## Discussion

Taken together, the convergent MRI findings supported the efficiency hypothesis, which predicted optimized transmission efficiencies as an underlying mechanism in WM neuroplasticity (Fig. 1). In particular, a common reduction in LFO signal amplitude supported the concept of improved synchronization, which would necessarily require increased transmission efficiency. As expected, WM fMRI activity was embedded within a wider network of distributed GM fMRI activity (Supplemental Fig. 1). By focusing on the direct evaluation of functional neuroplasticity in WM, it was possible to characterize the structural–functional changes between two established ROIs: the internal capsule (corticospinal tract) and the corpus callosum (interhemispheric transfer). Importantly, commonalities (and some differences) were clearly identified between the ROIs as a function of experimental changes in WM neuroplasticity. The findings were interpreted within the context of the two central aims:

*Aim 1*: Improved WM fMRI sensitivity: convergent fMRI evidence of functional neuroplasticity:

The findings confirmed that it was possible to improve WM fMRI sensitivity through integration of multiple MRI measures (Fig. 1 Panel A). Specifically, FCT analyses expanded the detection of neuroplasticity effects from the internal capsule to the corpus callosum. Importantly, LFO analyses confirmed common neuroplasticity effects across both ROIs (Fig. 1 Panel B). While LFOs converged (described below), the pattern of results showed that the two techniques were differentially sensitive to directional effects supporting the need for enhanced characterization across multiple MRI measures (Fig. 2).

While the current study replicated pre-post motor performance improvements across different MRI measures (Reid et al. 2017; Sale et al. 2017), the major novel finding was direct WM fMRI evidence of neuroplasticity. This finding was underpinned by common LFO signal changes across the ROIs in spite of differing sensitivities between DTI, WM HRF, and FCT functional measures. The frequency band containing the task frequency (~ 0.02 Hz, belonging to band A) exhibited a significant difference between LFO amplitude from baseline to endpoint. This drop was consistent with the effect seen across all bands of interest and in both ROIs (Fig. 1. Panel B).

Common LFO signal amplitude reductions were consistent with prior evidence of increased transmission efficiency (Bonzano et al. 2008; Jiang et al. 2019; Faragó et al. 2019; Sarma et al. 2021; Jiang et al. 2021). In particular, Bonzano et al. (2008) suggested that abnormal functional signal transmission across the corpus callosum was correlated to reduced motor coordination performance. The authors proposed that motor ability is dependent on white matter signal efficiency, which has been interpreted as myelination changes. The ability to directly measure functional neuroplasticity in WM is consistent with emerging evidence of important variability in HRFs across WM tissue (Li et al. 2019, 2021; Frizzell et al. 2020; Wang et al. 2020a,b).

The internal capsule is a key part of the corticospinal tract, which is critical for motor function and has been previously identified in WM fMRI studies (Frizzell et al. 2020; Gawryluk et al. 2011a,b; Maldjian et al. 1999; Mazerolle et al. 2013). In terms of fMRI methods, LH > RH LFO results corresponded with the LH > RH behavioral improvements in motor performance (Fig. 2 Panel C). While correlational analyses showed comparable internal capsule intra-correlations at both baseline and endpoint, there was a notable reduction in correlated LFO activity between the internal capsule and the corpus callosum at endpoint (Fig. 3).

The drop in cross-correlations between ROIs reflects reduced synchronization between brain areas. While speculative, the reduced synchronization may have resulted from reduced reliance on interhemispheric activity during the consolidation of motor learning. Supporting this, we reported a similar pattern of results in a recent clinical neuroimaging case study on recovered motor function following severe traumatic brain injury (Fickling et al. 2020), which corresponded with the growing stroke rehabilitation literature on the involvement of transcallosal activity during motor function recovery (Dodd et al. 2017).

The body of the corpus callosum is critical for motor coordination and has been previously reported in WM fMRI studies on inter-hemispheric transfer and motor response function (Caillé et al. 2005; Fabri 2014; Fabri and Polonara 2013; Fabri et al. 2011; Wang et al. 2020a,b). In the corpus callosum ROI, the FCT results also corresponded with LH > RH changes as measured by behavioral performance (Fig. 2 Panel B). Non-dominant hand performance improvements showed significantly greater reliance on interhemispheric support, which was further supported by GM fMRI activity findings (Supplementary Fig. 2, Panel A). However, the asymmetry appeared to be reversed for the dominant hand, with significant increases in R > L LFO amplitudes. Considering prior evidence that LFO amplitude increases are associated with reduced activity levels, the results indicated reliance on inter-hemispheric transfer for motor coordination. Again, supporting this interpretation, there was a corresponding drop in contralateral left hemisphere cortical GM fMRI activation in the precentral gyrus (Supplemental Fig. 2, Panel B).

The common LFO sensitivity to functional neuroplasticity, together with the pattern of changes in LFO amplitudes between ROIs, highlighted the potential role of ALFF as a metric of WM efficiency. Indeed, the consistent trends across ALFF frequency bands suggested WM-specific changes, consistent with prior ALFF WM reports (Jiang et al. 2019). Sarma et al. (2021) recently reported higher ALFF in WM for perinatally HIV-infected youth compared to controls. Jiang et al. (2021) also recently reported that healthy controls had lower ALFF than schizophrenic patients in the internal capsule and corpus callosum, which was associated with increased network disruption in the patient group. Studies comparing WM and GM LFOs have reported significant differences between frequency bands (Zuo et al. 2010; Biswal et al. 1995; Mather and Nga 2013). Given known differences in neuroplasticity between WM and GM, corresponding ALFF GM/WM differences and the consistent ALFF reduction across frequency bands provides additional support for common underlying mechanisms, such as the previously identified changes in myelination (Reid et al. 2017; Sale et al. 2017; Sampaio-Baptista and Johansen-Berg 2017).

*Aim 2*: functional MRI changes can be linked back to DTI structural changes:

Importantly, the findings confirmed preliminary structural–functional links between DTI and fMRI measures of neuroplasticity (Figs. 1, 2, and 4). Linking the WM fMRI changes back to DTI structural changes is critical to the efficiency hypothesis. The main findings revealed that DTI FA was correlated with BOLD signal synchronicity in the internal capsule (Fig. 1. Panel A). In this case, the direction of neuroplasticity change relative to performance improvements was an important factor. As expected, there were significant FA gains in the contralateral internal capsule, which corresponded with the behavioral non-dominant (left) greater than dominant (right) asymmetry. This pattern was consistent with increased transmission efficiency in the corticospinal tract.

While there were also DTI changes in the corpus callosum as a function of neuroplasticity, the patterns were more complex (consistent with the above results). Significantly higher correlations in FA and FCT were detected for both non-dominant (left) and dominant (right) hand performance (Fig. 4). For non-dominant hand performance, increased FA correlations likely reflected the parallel increase in LH > RH for LFO and FCT during increased transmission efficiency, consistent with the DTI LH > RH (Fig. 2A). For dominant hand performance, increased DTI-FA correlations likely reflected the reduced reliance on interhemispheric transmission (discussed above). Overall, the multimodal MRI findings supported common underlying structural mechanisms driving white matter functional neuroplasticity.

Neural activity is intrinsically driven by the anatomical architecture and connections present in the brain structure (Pernice et al. 2011). Mazerolle et al. (2010) demonstrated that function in the corpus callosum could be co-localized with DTI tractography seeded from regions of GM activity.

FCTs have successfully been used to model long-range WM tracts during resting state fMRI (Ding et al. 2016). The current finding showed significantly stronger correlations between FCT and DTI may provide increased sensitivity to WM functional changes, independent of estimating the hemodynamic response functions. Once such a relationship is established, the specific microstructural changes involved in white matter efficiency may be indicated by LFOs, in particular ALFFs, to evaluate the direction of changes in tract myelination.

### Caveats

There are at least three caveats to be considered. First, ROI selection for the left hemisphere was based on assumptions of symmetry. Given this assumption, it is possible that some WM effects in the left hemisphere were undetected. Second, there is increasing work that is better characterizing the WM HRF (Li et al. 2019, 2021; Courtemanche et al. 2018; Wang et al. 2020a,b) and improved approximations of the WM HRF will most likely improve sensitivity to WM effects. Finally, third, the longitudinal nature of the current study inherently required a smaller overall sample size. Following the initial demonstration, WM structural and functional change sensitivities can be further improved using larger sample sizes, particularly through existing databases.

Nonetheless, the findings supported the hypothesis of convergent neuroplasticity effects in the WM ROIs, with common LFO signal amplitude reductions lending strong support to underlying myelin-based transmission efficacies. Future work should investigate the microstructural changes detected using myelin water imaging to further link the functional results to the driving structural factors. Tractography based analyses may also further support our understanding of the changes in effect that underlie white matter fMRI activity modulation as measured by HRFs, LFOs, and FCT.

## Supplementary Information

Below is the link to the electronic supplementary material.

Supplementary file1 (PDF 165 kb)

## Declarations

### Code availability

Patch-based Functional Correlation Tensor code can be retrieved from: https://github.com/zyjshmily/ts-PFCTs (Zhou et al. 2017).

### Data availability

The datasets generated during and analyzed during the current study are not publicly available due them containing information that could compromise research participant consent but are available from the corresponding author on reasonable request.
