# Supplementary material for: Imaging functional neuroplasticity in human white matter tracts
Source: Brain Struct Funct. 2021 Nov 23;227(1):381–92. doi: 10.1007/s00429-021-02407-4 (PMC8741691; doi:10.1007/s00429-021-02407-4)
Supplement: Supplementary file 1 — Supplementary file1 (PDF 165 kb) [file 429_2021_2407_MOESM1_ESM.pdf]

Supplemental Figures

GM analysis:

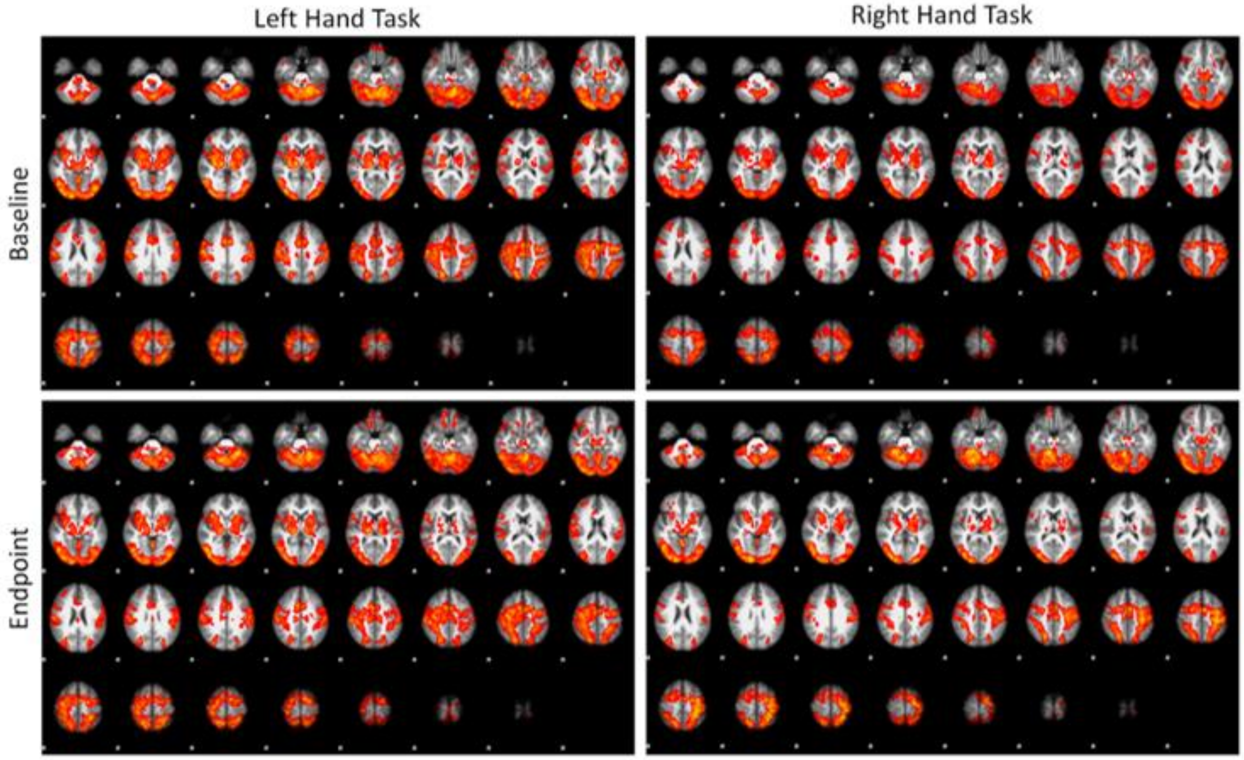

Supplementary Figure 1: Group level activation for each timepoint for each task condition ($z > 2.5$, $p < 0.05$, two-tailed, FWE).

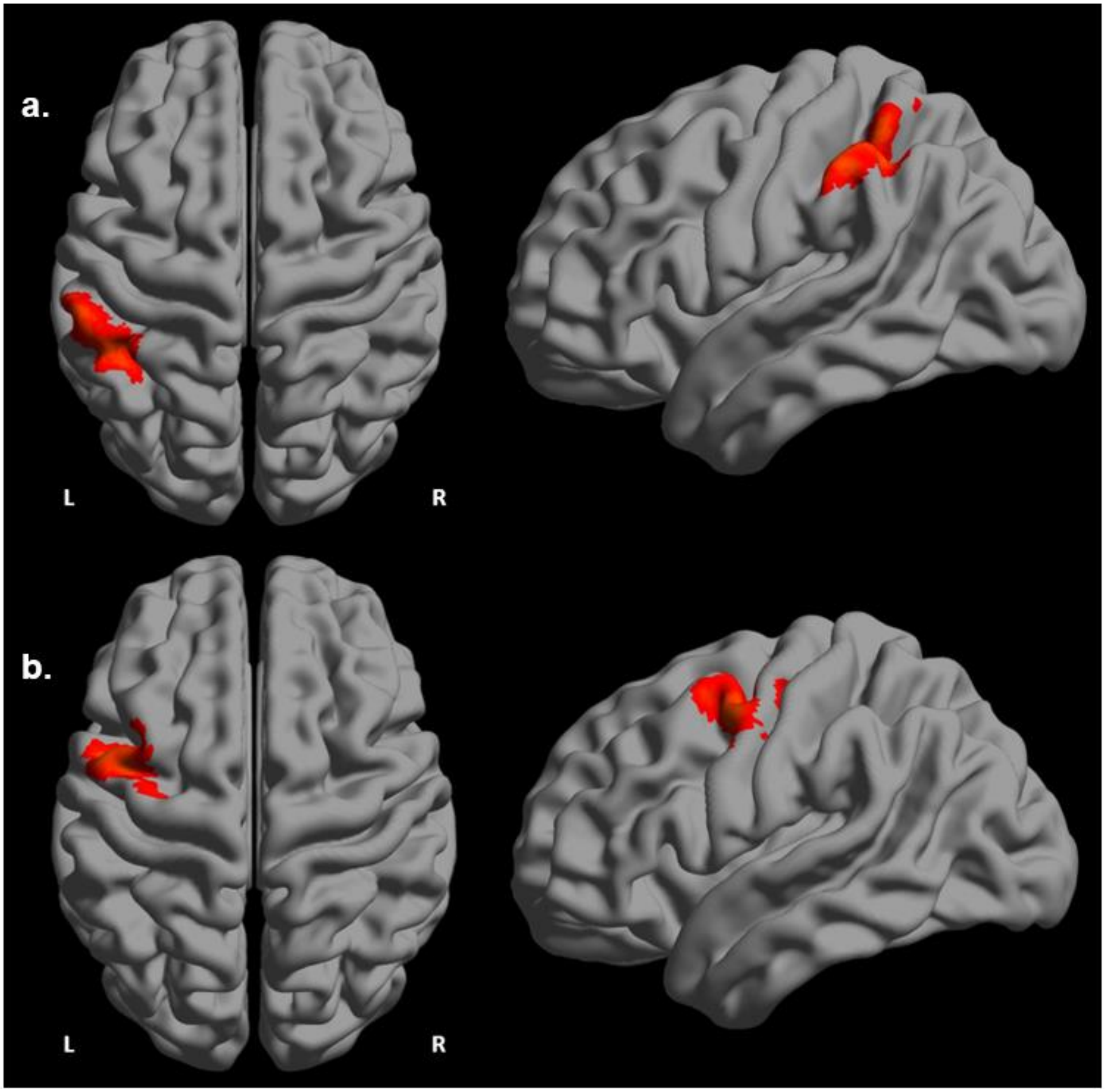

Supplementary Figure 2: Panel A - Group level difference for Baseline > Endpoint ($p < 0.05$, two-tailed, $z > 3.1$, FWE). Activation decreased in the left inferior parietal lobule for the left hand visuomotor task. Computed using FSL 6.0.0 Glm function using the canonical HRF. Panel B - Group level difference for Baseline > Endpoint ($p < 0.05$, two-tailed, $z > 3.1$, FWE). Activation decreased in the left precentral gyrus for the right hand visuomotor task. Computed using FSL 6.0.0 Glm function using the canonical HRF.
